# Enzalutamide-associated acute exacerbation of interstitial lung abnormality: a case report

**DOI:** 10.3389/fmed.2026.1861913

**Published:** 2026-07-07

**Authors:** Siji Meiduo, Yu Deng, Yulai Yuan, Feng Yang, Ling Lin, Tao Zhang, Suhua Ao

**Affiliations:** 1 Southwest Medical University, Luzhou, China; 2 The Affiliated Traditional Chinese Medicine Hospital, Southwest Medical University, Luzhou, China

**Keywords:** acute exacerbation, enzalutamide, interstitial lung abnormality, monitor, nintedanib, prostate cancer

## Abstract

Enzalutamide, a second-generation androgen receptor inhibitor (SGARI) for prostate cancer, is associated with rare but severe pulmonary toxicities. While typically manifesting as drug-induced interstitial lung disease (DI-ILD), these agents can also trigger acute exacerbation or rapid progression of pre-existing interstitial lung abnormalities (ILA). We report the case of an 83-year-old man with baseline ILA and emphysema who had previously undergone a laparoscopic radical prostatectomy. He maintained stable respiratory symptoms on bicalutamide for four years but developed progressive dyspnea within one month of switching to enzalutamide for biochemical recurrence. However, a chest computed tomography (CT) scan was not performed promptly upon symptom deterioration. This missed opportunity for early radiologic evaluation resulted in a 10-month diagnostic delay. Following the immediate discontinuation of enzalutamide and the concurrent initiation of nintedanib, the patient achieved radiologic stability and demonstrated improved exercise tolerance at the 6-month follow-up. From the patient’s perspective, the rapid progression of dyspnea was highly distressing, but he expressed significant relief and satisfaction with the improvement in his exercise tolerance following the medication adjustment. This clinical course highlights that prompt withdrawal of the offending agent is paramount, while phenotype-driven antifibrotic therapy may serve as a potential supportive intervention once irreversible fibrosis is established.

## 1 Introduction

Enzalutamide significantly improves survival outcomes in metastatic castration-sensitive and castration-resistant prostate cancer as a second-generation androgen receptor inhibitor (SGARI). However, it carries a potential risk of pulmonary toxicity, particularly drug-induced interstitial lung disease (DI-ILD). While SGARI-ILD cases have been increasingly valued internationally, the existing literature predominantly focuses on apalutamide. Consequently, clinical vigilance regarding enzalutamide-associated pulmonary toxicity remains remarkably limited.

Furthermore, current pharmacovigilance data indicate a strong geographical clustering of SGARI-induced ILD in Asian countries, particularly Japan, suggesting a potential genetic predisposition (1–5). Despite this, to the best of our knowledge, no confirmed cases of enzalutamide-associated pulmonary fibrosis have been reported in China. This report presents the first documented case in a Chinese patient, featuring a clinical course where the failure to perform a prompt chest CT scan upon respiratory deterioration contributed to a delayed recognition of the acute exacerbation of underlying interstitial lung abnormalities (ILA), ultimately leading to irreversible fibrosis. We aim to enhance clinical vigilance, highlight diagnostic pitfalls, and discuss targeted therapeutic strategies.

## 2 Manuscript formatting

### 2.1 Case presentation

An 83-year-old male underwent laparoscopic radical prostatectomy in 2019 for prostate cancer with a Gleason score of 9 (5 + 4). Postoperatively, he remained stable on bicalutamide and goserelin for four years. In August 2024, due to biochemical recurrence with rising prostate-specific antigen (PSA) levels, his doctor switched bicalutamide to enzalutamide. Within one month of initiating enzalutamide, the patient experienced worsening cough and exertional dyspnea; however, a chest CT was not performed at that time, and these early symptoms were unfortunately overlooked without further radiologic investigation. Over the subsequent 10 months, his respiratory symptoms gradually progressed. Ultimately, he was admitted to the respiratory department with a significantly impaired six-minute walk distance (6MWD) of 180 meters. Physical examination revealed a respiratory rate of 25/min at rest, cyanotic lips, a barrel-shaped chest and bilateral Velcro crackles. There were no signs of advanced chronic hypoxia such as digital clubbing, nor clinical signs suggestive of connective tissue disease, such as joint deformities, mechanic’s hands, sclerodactyly, or rheumatoid nodules. He had a 10-year history of emphysema and a 30-pack-year smoking history but had quit 10 years prior, and previously worked as an office clerk, with no environmental or occupational exposures to birds, pesticides, or organic dusts (e.g., mushrooms). He had no family history of interstitial lung disease, autoimmune disorders, or known genetic predispositions to pulmonary fibrosis. A retrospective review of prior chest computed tomography (CT) scans revealed pre-existing baseline interstitial abnormalities. The 2020 CT showed very mild subpleural interstitial abnormalities in the basal segment of the left lower lobe (Figure 1A). By 2024, bilateral subpleural fine reticulation, ground-glass opacities, and traction bronchiolectasis were visible, meeting the definition of ILA and appearing mildly progressive compared with 2020 (Figure 1B). At this time, the echocardiogram showed no abnormalities. However, the chest CT scan upon admission in July 2025 revealed a marked progression to extensive bilateral interstitial fibrosis. Radiographically, the lesions were characterized by coarse reticulation and traction bronchiectasis, exhibiting a clear subpleural and lower-lobe predominance. Notably, active inflammatory signs such as diffuse ground-glass opacities (GGOs) were absent, indicating a transition to an irreversible fibrotic phenotype. Concurrently, the scan demonstrated background bilateral emphysema accompanied by pulmonary bullae, scattered minor calcifications, and mild tracheal mucous plugging. Furthermore, cardiovascular evaluation of the CT images highlighted prominent dilatation of the pulmonary artery and cardiomegaly (Figure 1C). These radiological findings of right heart strain were concomitantly confirmed by echocardiography, which demonstrated secondary pulmonary hypertension with an estimated systolic pressure of 46 mmHg (Figure 2). Pulmonary function tests showed no obstructive ventilatory defects, but a moderately reduced diffusing capacity for carbon monoxide (Table 1). Arterial blood gas analysis revealed an oxygenation index (PaO_2_/FiO_2_) of 280.8 mmHg. Autoimmune and infectious agents were unremarkable. Following a multidisciplinary team (MDT) discussion that systematically ruled out other common etiologies, it was concluded that the patient experienced an enzalutamide-associated acute exacerbation or acceleration of pre-existing ILA, leading to rapid fibrotic progression. Therefore, enzalutamide was discontinued, with anticancer therapy maintained on goserelin alone. Nintedanib was promptly initiated as antifibrotic therapy. Six months later, follow-up evaluation showed radiologic stability on CT and a modest improvement in DLCO (Figure 1D; Table 1). Notably, his 6MWD increased to 260 meters, objectively confirming the enhancement in his exercise tolerance. Significant events throughout the patient’s illness are highlighted in the line (Figure 3).

**Figure 1:**
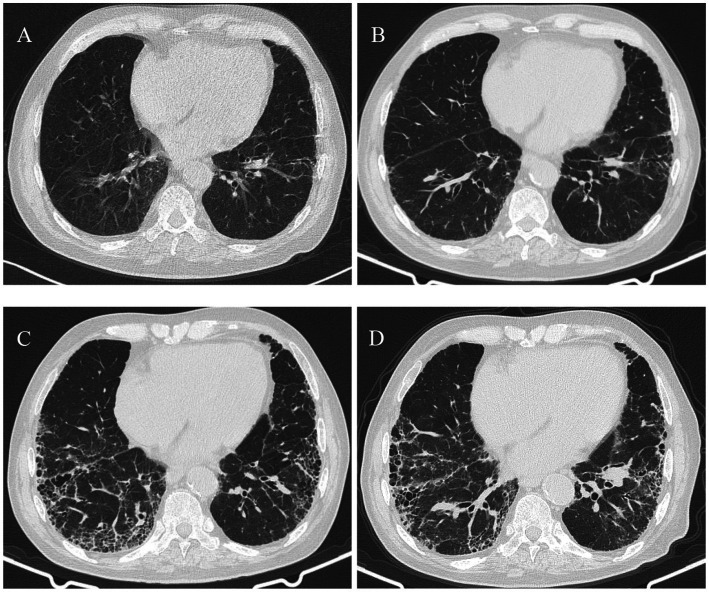
Chest CT evolution associated with androgen receptor inhibitor therapy. **(A)** (2020): Emphysema and mild subpleural interstitial abnormalities. **(B)** (On bicalutamide therapy for four years, 2024): Mildly progressive bilateral subpleural fine reticulation and ground-glass opacities, consistent with ILA. **(C)** (On enzalutamide therapy for ten months, 2025): bilateral interstitial fibrosis. **(D)** (Post-antifibrotic therapy, 2026): Radiologic stability with no further progression.

**Figure 2:**
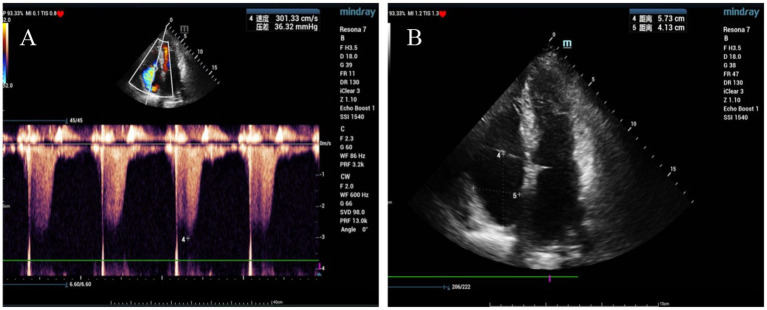
Echocardiographic evidence of secondary-pulmonary hypertension in 2025. **(A)** Continuous-wave Doppler: TR Vmax 3. 01 m/s, ΔP 36 mmHg; estimated sPAP 46 mmHg, indicating mild-to-moderate PH. **(B)** Apical 4-chamber view: RV longitudinal diameter 5. 73 cm (>upper limit 5. 5 cm), consistent with chronic pressure overload.

**Table 1:** Serial pulmonary function tests in a patient with enzalutamide-associated rapid progression of pulmonary fibrosis before and after initiating nintedanib.

Pulmonary function parameters	2025		2026	
	Measured value	% Pred	Measured value	% Pred
FVC (L)	2.94	92%	3.22	100%
FEV1(L)	2.76	117%	2.61	111%
FEV1/FVC	93.95	129%	80.96	112%
DLCO	2.95	41%	3.15	44%
VA(L)	4.59	74%	4.65	75%
TLC(L)	4.75	75%	4.80	76%

**Figure 3:**
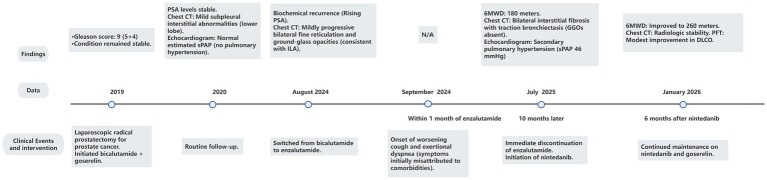
Timeline of the patient’s clinical course, diagnostic findings, and therapeutic interventions.

## 3 Discussion

The 10-month delay in recognizing this patient’s symptoms led to advanced clinical findings, including irreversible interstitial fibrosis and pulmonary hypertension. To establish this probable diagnosis, our MDT—comprising urologists, pulmonologists, and thoracic radiologists—systematically evaluated and excluded alternative etiologies that could mimic progressive fibrosing ILD. First, connective tissue disease-associated ILD (CTD-ILD) was confidently ruled out due to the complete absence of specific extrapulmonary manifestations and an unremarkable comprehensive autoimmune panel, including negative antinuclear antibodies (ANA) and myositis-specific autoantibodies. Second, we carefully considered smoking-related ILD (SR-ILD) and the natural progression of Combined Pulmonary Fibrosis and Emphysema (CPFE), given the patient’s significant smoking history and baseline emphysema. However, the natural history of these conditions is typically indolent and slowly progressive over years; they cannot account for the abrupt and severe deterioration of respiratory symptoms that emerged within exactly one month of initiating enzalutamide. Third, a diagnosis of typical Idiopathic Pulmonary Fibrosis (IPF) was excluded because the baseline 2020 and 2024 CT scans lacked the characteristic widespread honeycombing of a definitive Usual Interstitial Pneumonia (UIP) pattern, and IPF rarely exhibits such a sudden, temporally drug-linked acceleration without a clear infectious or hemodynamic trigger. While histopathological confirmation via bronchoalveolar lavage (BAL) or surgical/transbronchial lung biopsy remains the gold standard for differentiating these etiologies, these invasive procedures were ultimately deferred. Given the patient’s advanced age (83 years) and his explicit preference to avoid invasive interventions, our MDT respected his personal wishes, thereby necessitating a pragmatic clinical-radiologic diagnosis.

Having systematically excluded these primary and secondary fibrosing lung diseases, we evaluated the probability of an adverse drug reaction. Notably, the patient had maintained stable respiratory symptoms during four years of prior bicalutamide therapy. Because existing literature indicates that DI-ILD predominantly occurs within the first year of treatment, this prolonged, symptom-free tolerance effectively excludes bicalutamide as the causative agent. In contrast, the abrupt onset of progressive dyspnea emerged precisely within one month of switching to enzalutamide. This tight temporal correlation perfectly matches the high-risk window for SGARI-associated pulmonary toxicity. Consequently, based on this definitive timeline, the exoneration of prior therapies, and the exclusion of alternative etiologies, the Naranjo Adverse Drug Reaction Probability Scale yielded a score of 7 points, formally identifying enzalutamide as the “probable” causative factor (6, 7). Beyond establishing causality, this specific clinical scenario warrants a deeper analysis of two critical aspects: the precise clinical-radiologic phenotype of this pulmonary toxicity, and the severe consequences of its delayed recognition.

Regarding the phenotypic characterization, a retrospective review of the patient’s radiological history fundamentally shifts the diagnostic paradigm from a *de novo* DI-ILD to a drug-triggered AE-ILA. This exhaustive process directly elucidates a fundamental conceptual boundary: while traditional *de novo* DI-ILD implies an idiosyncratic toxic injury arising in previously pristine lung parenchyma, current consensus defines ILA as incidental, non-dependent CT abnormalities denoting a highly vulnerable lung parenchyma with a recognized risk of progressive fibrosis (8). In our patient, the transition from mild subpleural abnormalities in 2020 to distinct reticulation and traction bronchiolectasis by 2024 established a baseline of fragile alveolar-immune homeostasis. In this specific context, the explosive onset of severe exertional dyspnea precisely one month after enzalutamide initiation suggests that the drug did not merely exert a slow, cumulative direct toxicity. Instead, we hypothesize that enzalutamide acted as a precipitating “second hit”—a molecular and immunological trigger that acutely disrupted the lung’s marginal baseline homeostasis, driving a rapid kinetic progression that strongly aligns with an acute exacerbation rather than a gradual toxic accumulation (9). Ideally, a CT scan at the precise time of symptom deterioration would have identified the acute development of bilateral ground-glass opacities superimposed on the background ILA. Although this critical acute-phase imaging was missed, the subsequent fibrotic pattern (Figure 1C) clearly reflects the devastating fibrotic remodeling that typically follows such an acute exacerbation. This critical distinction underscores a profound clinical warning: SGARIs possess the dual potential to not only induce primary lung injury but also to act as a fulminant catalyst in lungs already harboring subclinical fibrotic changes, rapidly accelerating the trajectory toward end-stage, irreversible fibrosis.

Clinically, this case highlights a critical diagnostic pitfall in geriatric oncology. The most plausible explanation for the 10-month diagnostic delay is that a chest CT was not performed promptly when the patient first developed respiratory deterioration, resulting in a missed opportunity to identify the acute exacerbation of his underlying ILA. In older adults, non-specific early respiratory symptoms like exertional dyspnea are easily overlooked or reflexively misattributed to physiological aging or general decline, delaying necessary imaging. Clinicians must maintain a high index of suspicion: any new-onset respiratory symptom within the first few months of SGARI initiation should be evaluated as potential drug-induced pulmonary toxicity, rather than reflexively attributed to the natural progression of geriatric comorbidities. As highlighted in systematic literature reviews of other targeted therapies, such as rituximab, non-specific early respiratory symptoms are frequently confounded by underlying comorbidities, leading to widespread diagnostic delays (10). This underscores that the symptom overlap and subsequent delayed recognition observed in our patient represent a clinical challenge in modern oncology.

The exact molecular mechanism by which enzalutamide triggers pulmonary toxicity remains elusive, though it may involve profound androgen receptor (AR) blockade impairing alveolar epithelial repair injury (11–14). Compared to older anti-androgens, the superior target engagement of SGARIs might explain this severe class effect (15). Clinically, while early-stage SGARI-ILD typically presents with inflammatory ground-glass opacities that are responsive to corticosteroids (16), our patient’s 10-month diagnostic delay allowed the disease to progress to an irreversible fibrotic phenotype. At this advanced stage, high-dose glucocorticoids were deemed unsuitable due to their diminishing returns for fibrotic remodeling and the elevated infection risk in geriatric patients (17). Instead, we initiated nintedanib (18). Although the prompt cessation of the offending agent likely played the predominant role in halting disease progression, nintedanib’s contribution to achieving radiologic stabilization highlights a potential phenotype-driven supportive strategy for established fibrosis.

Beyond individual management, current pharmacovigilance data indicate a geographical clustering of SGARI-associated ILD in Asian populations, suggesting a potential genetic predisposition (19). Consequently, heightened clinical vigilance is strongly warranted in these populations. To avoid the severe diagnostic pitfalls encountered in this case, we advocate for a baseline pulmonary assessment (HRCT and DLCO) prior to SGARI initiation, followed by symptom-directed monitoring—such as incorporating the 6MWD test to detect subtle exertional desaturation. If such monitoring confirms SGARI-associated pulmonary toxicity, the offending agent must be discontinued immediately. Furthermore, switching to alternative SGARIs is strongly discouraged due to the high risk of class-effect cross-toxicity. Subsequent antineoplastic regimens must then be carefully individualized by the oncology MDT, prioritizing alternative options without overlapping lung toxicities to protect the patient’s fragile respiratory reserve.

A primary strength of this case report is the comprehensive four-year longitudinal radiologic tracking, which enabled the retrospective identification of baseline ILA and the subsequent enzalutamide-triggered exacerbation. Furthermore, the MDT approach highlights a pragmatic and safe diagnostic pathway in geriatric oncology. The main limitation is the lack of histopathological confirmation via lung biopsy; however, as previously discussed, this was a conscious clinical decision made to respect the elderly patient’s preference and avoid prohibitive procedural risks. Additionally, the absence of a chest CT precisely at the onset of respiratory deterioration limited our ability to capture the acute inflammatory phase.

## 4 Conclusion

In conclusion, this first documented case of enzalutamide-associated rapid progression of pulmonary fibrosis in China highlights a rare but serious pulmonary toxicity that can be easily obscured by geriatric comorbidities and pre-existing lung disease. The prolonged diagnostic delay observed in our patient underscores the clinical imperative for proactive respiratory monitoring—specifically incorporating baseline HRCT, DLCO, and routine 6MWD assessments—when initiating SGARI therapy. Furthermore, while the immediate cessation of enzalutamide was the critical intervention to halt disease progression, the patient’s subsequent stabilization alongside nintedanib administration highlights the clinical value of phenotype-driven management. This combined approach suggests a potentially viable therapeutic option when irreversible fibrotic changes render traditional corticosteroid therapy unfavorable. As the clinical application of enzalutamide continues to expand, heightened vigilance and tailored multidisciplinary care remain essential to recognize this adverse event early and mitigate severe pulmonary outcomes, particularly in potentially susceptible Asian populations.

## Data Availability

The original contributions presented in the study are included in the article/Supplementary material, further inquiries can be directed to the corresponding author/s.
