# Supplementary figures and images for: Enzalutamide-associated acute exacerbation of interstitial lung abnormality: a case report
Source: Front Med (Lausanne). 2026 Jul 7;13:1861913. doi: 10.3389/fmed.2026.1861913 (PMC13385288; doi:10.3389/fmed.2026.1861913)

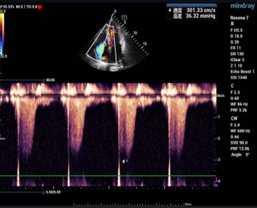

Supplement: Supplementary file 1 [file Image_1.JPEG]

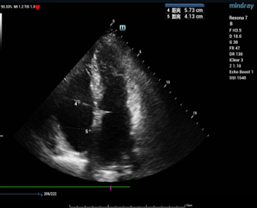

Supplement: Supplementary file 2 [file Image_2.PNG]

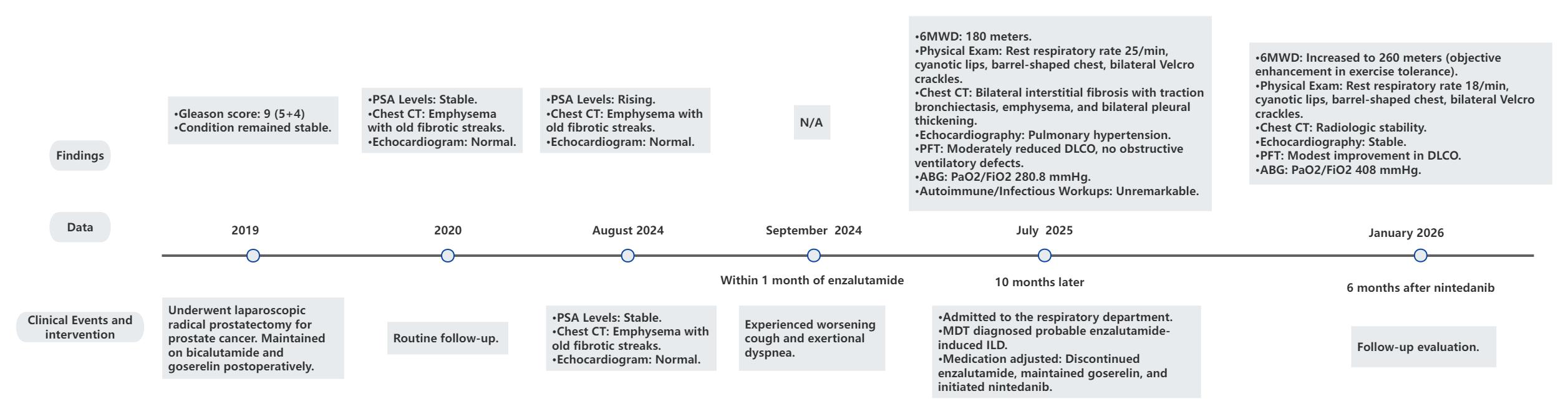

Supplement: Supplementary file 3 [file Image_3.JPEG]
